# On the Actinometric Measurement of Absolute Luminescence Quantum Yields*

**DOI:** 10.6028/jres.080A.041

**Published:** 1976-06-01

**Authors:** J. N. Demas, B. H. Blumenthal

**Affiliations:** Chemistry Department, University of Virginia, Charlottesville, Virginia 22901

**Keywords:** Absolute yield, chemical actinometry, correction factors, luminescence, quantum-flat actinometer, quantum yield

## Abstract

The theory of the measurement of luminescent quantum yields using chemical actinometry is described. The sample’s emission intensity is measured by nearly completely surrounding the sample with an actinometer solution, and the excitation intensity is directly measured with the same type of actinometer. The ratio of the measured sample emission intensity corrected for the fraction escaping through the excitation ports to the measured excitation intensity is the absolute luminescence yield. Equations, a suitable cell design, and computer calculated correction factors for different cell dimensions and optical densities are given. The absolute yield of the actinometer is not needed, only its relative response with wavelength. New quantum-flat actinometers which should greatly simplify the measurements are described.

## 1. Introduction

The measurement of absolute luminescence quantum yields is an important but experimentally difficult area [1, 2, 3].^1^ Most yield measurements are made relative to a luminescence standard rather than by an “absolute” method. It is thus imperative for good accuracy that the luminescent standards be accurately calibrated. To date, these standards have been derived almost exclusively from calorimetry or by reference to a standard scatterer, usually with a luminescent quantum counter detector.

The history of quantum yield measurements has demonstrated quite painfully that it is exceedingly difficult to detect and eliminate all systematic errors. Thus, materials selected for standards should be tested by as many independent and presumably reliable “absolute” methods as possible and rechecked as new techniques become available.

We describe here the theory of a conceptually new “absolute” method for measuring luminescence quantum yields based on chemical actinometers. The technique avoids many of the intrinsic error sources of the other methods and thus promises to be a useful check on existing and new standards. The current availability of numerous lasers with both high intensites and a wide range of wavelengths ranging from < 250 nm to > 800 nm coupled with new broadband, quantum-flat actinometers make the actinometric method most attractive. In addition to the theory we present a suitable cell design, tabular correction factors, and describe suitable actinometers.

## 2. Theory

The actinometric approach for determining yields measures the excitation intensity and the sample emission intensity by chemical actinometry; the ratio of the emission to excitation intensity, both corrected for the fraction of the excitation beam absorbed, is the absolute quantum yield. The total emitted intensity is measured by nearly completely surrounding the sample with the actinometer solution except for a small excitation port and correcting for the small port losses. The same type of actinometer is then used to measure the excitation beam intensity.

For the actinometer monitoring the emission intensity of the luminescent unknown, the amount of reaction in the actinometer, *D_x_* (mol of product), is given by

$$\begin{array}{l} D_{x}=I_{o}t_{x}\emptyset F_{E}^{\prime }{\bar{\theta }}_{x}\\ F_{x}=T\left(1-10^{-A_{x}}\right) \end{array}$$ (1)

where *I*_0_ (einstein/s) is the incident excitation intensity, *t_x_*(s) is the irradiation time, *F_x_* is the fraction of incident excitation light absorbed by the unknown, *T* is the effective transmittance of the entrance window, *A_x_* is the absorbance of the solution to the excitation beam, ∅ is the sample’s absolute luminescence efficiency, $F_{E}^{\prime }$ is the fraction of emitted light captured by the actinometer, and ${\bar{\theta }}_{x}\left(\text{mol}/\text{einstein}\right)$ is the actinometer’s effective photochemical quantum yield for the emission band. It is assumed that the excitation beam is monochromatic, all of the emission passing into the actinometer is absorbed, and reabsorption-reemission corrections are negligible. ${\bar{\theta }}_{x}$ is given by

$${\bar{\theta }}_{x}=\frac{\int_{0}^{\infty }F\left(\bar{\nu }\right)\theta_{x}\left(\bar{\nu }\right)d\bar{\nu }}{\int_{0}^{\infty }F\left(\bar{\nu }\right)d\bar{\nu }}$$ (2)

where $\bar{\nu }$ is energy in cm^−1^, $F\left(\bar{\nu }\right)$ (relative quanta/cm^−1^ of bandwidth) is the corrected relative emission spectrum and $\theta_{x}\left(\bar{\nu }\right)$ (mol/einstein) is the variation of the actinometer yield with excitation energy.

In the measurement of the excitation beam intensity, the amount of reaction in the actinometer, *D_s_*(mol), is given by

$$\begin{array}{c} D_{s}=I_{0}t_{s}F_{s}\theta_{s}\\ F_{s}=T\left(1-10^{-A_{s}}\right) \end{array}$$ (3)

where *t_s_*(s) is the irradiation time, *F_s_* is the fraction of the excitation beam absorbed by the actinometer, *T* is the same as eq 1,*A_s_* is the absorbance of the actinometer solution to the laser, and θ*_s_* (mol/einstein) is the actinometer’s yield at the excitation wavelength. The absolute luminescence quantum yield is then given by

$$\emptyset =\left(\frac{D_{x}}{D_{s}}\right)\left(\frac{t_{s}}{t_{x}}\right)\left(\frac{1-10^{-A_{s}}}{1-10^{-A_{x}}}\right)\left(\frac{\theta_{s}}{{\bar{\theta }}_{x}}\right)\left(\frac{1}{{F^{\prime }}_{E}}\right).$$ (4)

The *D*’s, *A’s* and *t*’s are directly measurable and $F_{E}^{\prime }$ can be evaluated from geometric considerations (see below). At first it might appear that this method can be no more accurate than the absolute accuracy of the evaluation of the actionometer’s yield, $\theta \left(\bar{\nu }\right)$, a process which is rarely good to better than 10 percent. In reality since eq 4 uses the ratio of θ*_s_* to ${\bar{\theta }}_{x}$, only the variation of $\emptyset \left(\bar{\nu }\right)$ with $\bar{\nu }$ need be known accurately. As long as data from the same workers are used, this error is likely to be substantially smaller than 10 percent and quite possibly less than 5 percent. Also, as we shall show, actinometers with intrinsically quantum-flat responses are becoming available which will make $\theta_{s}/{\bar{\theta }}_{x}=1.000$ within ~1–2 percent, regardless of how accurately the absolute yield is known.

### The Model

**Figure 1:**
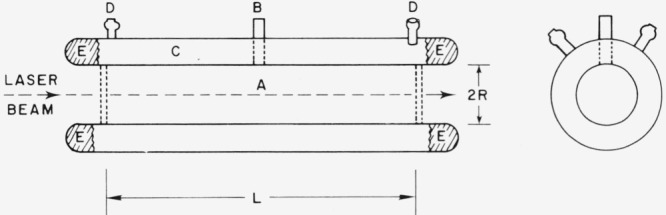
Cell design for luminescence quantum yield measurements by actinometry. a. Luminescent sample. b. Filling stem for sample. c. Actinometer solution compartment. d. Filling stems for actinometer which are also suitable for a flow actinometer system. e. Silvering on actinometer jacket ends to reflect emission transmitted down glass walls back into solution. To extend the maximum wavelength of the actinometer, the entire actinometer jacket could be silvered to double the absorption path for emission.

Figure 1 shows an easily fabricated cell suitable for measuring absolute yields by actinometry. The cell, built much like a reflux condenser, has a long, central irradiation volume with a small diameter filling stem. The outer jacket contains the actinometer solution which intercepts and absorbs a large fraction of the emitted light. The two filling ports on the actinometer jacket facilitate filling and permit the use of flow actinometers.

This cell design has numerous advantages. The system is only suitable for use with laser excitation; therefore, the monochromatic laser light eliminates, in virtually all solution cases, the need for effective absorbance corrections arising from variation of absorbance over the excitation band [4]. Questionable refractive index corrections are also eliminated. The high symmetry and entering and exiting excitation ports simplify evaluation of *F_x_* and $F_{E}^{\prime }$; further, the exit port removes unabsorbed excitation light from the system so that it cannot affect the actinometer. By silvering the ends of the actinometer jacket, radiation light piped down the glass walls can be directed back into the actinometer. By making *L*/*R* large, *F*′*_E_* can be made to approach unity as closely as desired. By choosing a large *L*/*R*, one can easily absorb a large fraction of the exciting light and still keep the reabsorption-reemission correction small. Thus, the system combines some of the best features of the optically dense and dilute approaches.

### Evaluation of $F_{E}^{\prime }$

To evaluate $F_{E}^{\prime }$ we make several assumptions, all of which will be quite accurate or will introduce negligible errors in a well-designed cell. These assumptions are: (1) the laser beam is centered and its diameter is small compared to *R*, (2) all emission not directly striking the windows is absorbed by the actinometer, (3) all of the emission transmitted by the cell windows is lost, (4) reabsorption-reemission corrections are negligible, and (5) the windows and cell walls are nonabsorbing.

*F*′*_E_* is divided into two terms, a geometric factor for direct capture of the emitted light and a correction for the emitted light reflected by the windows back into the cell which is subsequently absorbed by the actinometer. $F_{E}^{\prime }$ is given by

$$F_{E}^{\prime }=F_{E}+\left(1-F_{E}\right)r_{eff}F_{RA}$$ (5)

where *F_E_* is the fraction of primary emission that would be absorbed if the windows were perfectly transparent with no reflection losses, *r_eff_* is the fraction of primary emitted radiation reflected back into the cell by the windows, and *F_RA_* is that fraction of this reflected radiation which is eventually absorbed by the actinometer, the remainder eventually escaping. *F_E_* can be evaluated by

$$\begin{array}{l} F_{E}=\left(\frac{1}{2B}\right)\int_{0}^{L}\left\{\cos\left[\arctan\left(\frac{R}{\ell }\right)\right]+\cos\left[\arctan\left(\frac{R}{\left(L-\ell \right)}\right)\right]\right\}Ad\ell \\ A=\frac{\epsilon C\exp\left[-\ln\left(10\right)\epsilon C\ell \right]}{\left\{1-\exp\left[-\ln\left(10\right)\epsilon C\ell \right]\right\}}\\ B=1-\exp\left[-\ln\left(10\right)\epsilon C\ell \right] \end{array}$$ (6)

where *ϵ* is the sample’s molar extinction coefficient at the excitation wavelength and *C* is the sample concentration. The first and second cosine terms account for the fraction of radiation striking the entrance and exit windows respectively as a function of position in the cell. The *A* term accounts for the decrease in emission intensity along the tube caused by absorption. The *B* term corrects for the total fraction of excitation light absorbed in the cell.

For a very large *L*/*R* and not too high an optical density, *F_E_* will approach unity. For extremely high optical densities, however, the emission front surfaces at the entrance window where half the radiation could escape, and *F_E_* approaches 0.5.

Equation 6 has no obvious analytical solution and was evaluated numerically using Simpson’s rule. Because of the discontinuities in the integrand at *ℓ* = 0 and *ℓ* = *L*, the evaluation limits were just set very near both windows. Initially calculations were done on a Hewlett Packard 2000^2^ system in time sharing BASIC, but its ~6 – 7 significant figures proved inadequate. All calculations presented here were done on a Hewlett Packard 9100 B programmable desk calculator which has 10–12 significant figures; there were no problems with convergence. Integration was performed over the range *ℓ*/*L* = 10^−5^ to 0.99999 with 200 subdivisions. Increasing the number of divisions to 1000 caused no changes in the fifth significant figure. Our calculated *F_E_*’*s* are thus accurate to better than 0.1 percent. Calculated results for *F_E_*’*s* as a function of *L*/*R* and *A_x_* are given in table 1.

## 3. Discussion

Table 1 reveals that, as expected, *F_E_* approaches unity for large values of *L*/*R* with low to intermediate values of *A_x_.* Also, for very large values of *A_x_, F_E_* seems to approach 0.5. *F_E_* decreases as *A_x_* increases because of the movement of the emission towards one end where the escape factor is greater.

As a practical consideration *F_E_* should be as close as possible to unity. Fortunately this is not difficult. Even for a cell which is only 2.5 times longer than its diameter (*L*/*R* = 5), *F_E_* is >75 percent for *A_x_* ⩽ 2, an acceptable value. More realistic values of *L*/*R* for actinometer cells would be 20–50 which yield *F_E_* > 90 percent for *A_x_* ⩽ 2.0. Even values of *L*/*R* of 100–200 are feasible; a 50 cm cell would be 0.5–1 cm in diameter; in these cases *F_E_* would exceed 90 percent for *A_x_ ⩽* 3. It is thus clear that excellent collection efficiency of the emission can be readily obtained.

In the evaluation of ∅, $F_{E}^{\prime }$ rather than *F_E_* is actually required. We have not done a quantitative analysis for $F_{E}^{\prime }$ because *r_eff_* and *F_RA_* are quite difficult to evaluate, but in a well-designed cell the error associated with replacing $F_{E}^{\prime }$ by *F_E_* is quite small. For example, in a cell with a large *L*/*R*, most of the emission incident on the cell windows will be at near normal incidence; thus, we can use *r_eff_* ~ 0.04 for a glass-air interface. For *F_E_* = 90 percent, $F_{E}^{\prime }$ will be <0.5 percent greater than *F_E_* and the error falls for larger *F_E_*’s.

Until recently the potential choices of actinometers were limited to ferrioxalate and Reinecke’s salt. The ferrioxalate actinometer will yield total absorption of the emitted radiation up to ~480 nm using a 5 cm thick actinometer solution (0.15 F). The yield is not strongly wavelength dependent from 254 nm to 480 nm, and calibration is sufficiently detailed to permit accurate evaluation of ${\bar{\theta }}_{x}$.

Reinecke’s salt offers much deeper red penetration, ~610 nm for 2 × 10^−2^*M* solution and a 5 cm minimum cell length. The yield is more nearly constant than ferrioxalate over the 390–620 nm range. Unfortunately Reinecke’s salt has serious disadvantages. The yield changes sharply below 390 nm, and detailed data in this region are lacking. The complex is difficult to dissolve at high concentrations and undergoes a relatively rapid thermal reaction (~0.6%/h). Although it is about an order of magnitude less sensitive than ferrioxalate, this is not likely to be a problem with a high intensity laser excitation source.

New photosensitized actinometers promise to eliminate the previous difficulties. For example, the tris-(2,2-bipyridine)ruthenium(II) photooxidation of tetramethylethylene has been developed as an actinometer for high power lasers [5]. The system should be intrinsically quantum flat, because the lowest excited state is responsible for the sensitization; luminescence experiments have verified that the efficiency of population of the emitting state is constant to ~±2 percent over this region [6]. A solution 10^−3^*M* in the ruthenium complex will absorb all radiation below ~520 nm in a 2 cm pathlength. By using similar osmium(II) complexes as the sensitizer [6, 7, 8], total absorption of the emission and a quantum flat response below ~700 nm should be realized. These systems use volumetric monitoring of the consumed O_2_ and are thus not very sensitive, but laser excitation supplies adequate intensity. Finally, because the consumed O_2_ must be replaced and inhomogeneity of the reactants can be a problem in a static system, these actinometers must be operated in a flow system.

An analogous system which also shows promise is the methylene blue sensitized photooxidation of tetramethylethylene. Solutions can easily be made totally absorbing to beyond 700 nm in a 1 cm pathlength, and the yield is comparable to the Ru(II) and Os(II) systems. The quantum yields may, however, not be perfectly flat, and minor corrections may be required.

In summary, we feel that the actinometric method, although too complex for routine measurements, will prove especially useful in developing primary luminescence quantum yield standards. This method eliminates most of the error sources inherent in other absolute techniques, and thus supplies a valuable check. The technique is currently feasible for compounds emitting below 520 nm, and with the natural evolution of actinometry, operation to 700 nm and beyond should soon be feasible.

## Figures and Tables

**Table 1:** Emission absorption fraction, *F_E_*

*L*/*R*	5	10	20	50	100	200
*A_x_*						
						
0.5	0.8141	0.9001	0.9479	0.9786	0.9892	0.9946
1.0	.7986	.8866	.9385	.9738	.9865	.9932
1.5	.7776	.8681	.9255	.9670	.9828	.9912
2.0	.7551	.8478	.9109	.9593	.9785	.9889
2.5	.7333	.8277	.8960	.9513	.9739	.9864
3.0	.7136	.8089	.8818	.9434	.9693	.9839
3.5	.6962	.7916	.8683	.9357	.9648	.9814
4.0	.6808	.7760	.8556	.9282	.9604	.9790
5.0	.6555	.7488	.8327	.9141	.9585	.9741
6.0	.6357	.7263	.8125	.9010	.9437	.9694
7.0	.6199	.7072	.7947	.8887	.9359	.9649
8.0	.6071	.6910	.7786	.8773	.9284	.9604
9.0	.5964	.6770	.7642	.8665	.9212	.9561
10.0	.5875	.6647	.7512	.8564	.9143	.9518

## Footnotes

1 Figures in brackets indicate the literature references at the end of this paper.

2 In order to describe materials and experimental procedures adequately, it is occasionally necessary to identify commercial products by manufacturer’s name or label. In no instance does such identification imply endorsement by the National Bureau of Standards, nor does it imply that the particular product or equipment is necessarily the best available for that purpose.
